# Mould Infection Simulating Pulmonary Tuberculosis

**Published:** 1915-11

**Authors:** 


**Mould Infection Simulating Pulmonary Tuberculosis.** August O. Truelove of Indianapolis, Ind. Med. Jour., July. Patient male, 47, worked in grist mill 12-25, butter-tub factory,

25-35, since then has been on dairy farms or driving milk and bread wagons, all occupations predisposing to dust. Sputum copious, tenacious, containing staphylacocci, streptococci and spores indentified as of aspergillus fumigatus. Sticker has collected 20 cases of mould infection of lung.

